# Supplementary figures and images for: Accurate wisdom of the crowd from unsupervised dimension reduction
Source: R Soc Open Sci. 2019 Jul 31;6(7):181806. doi: 10.1098/rsos.181806 (PMC6689600; doi:10.1098/rsos.181806)

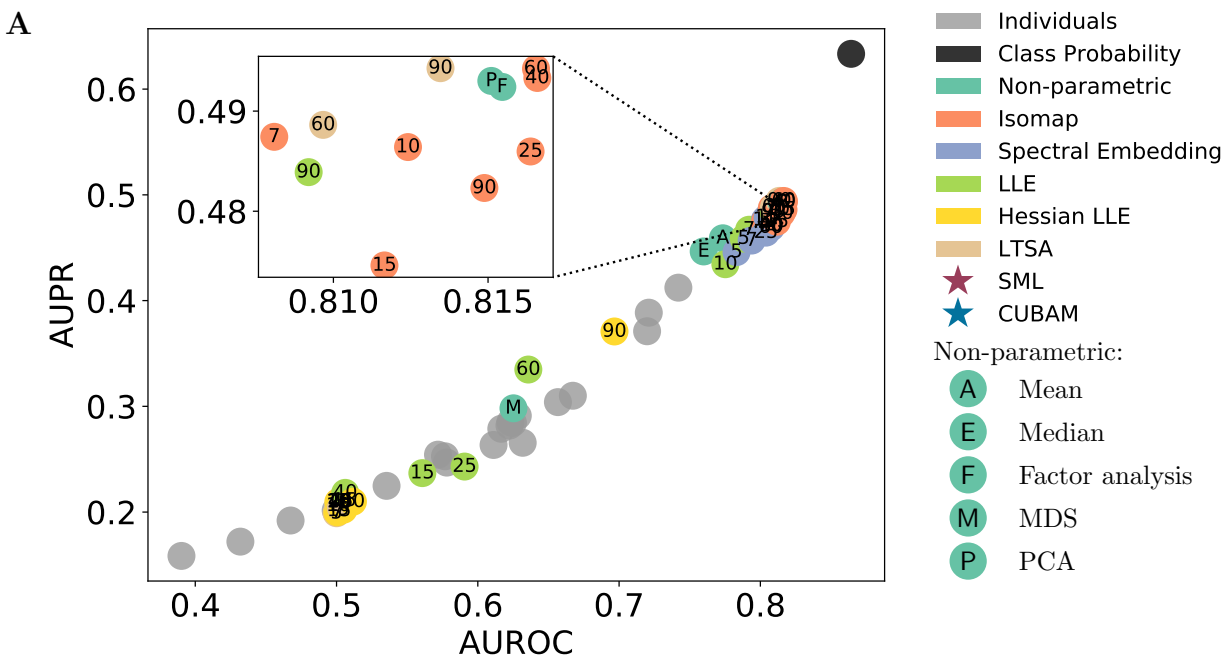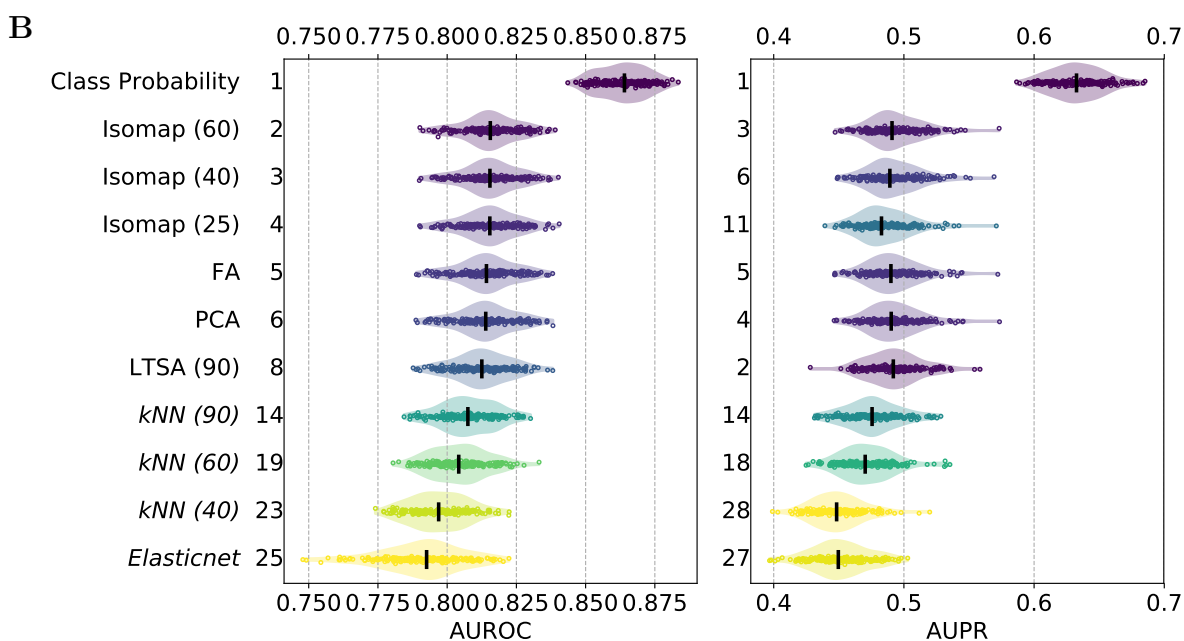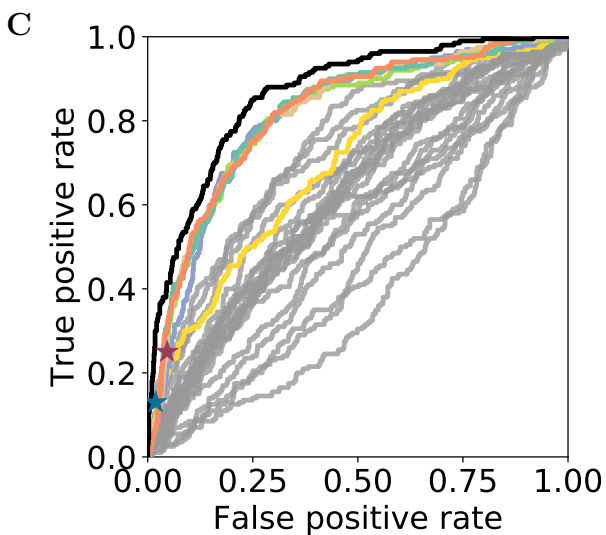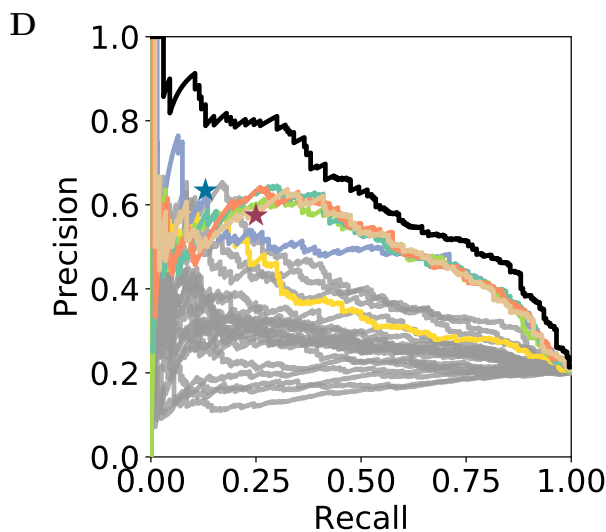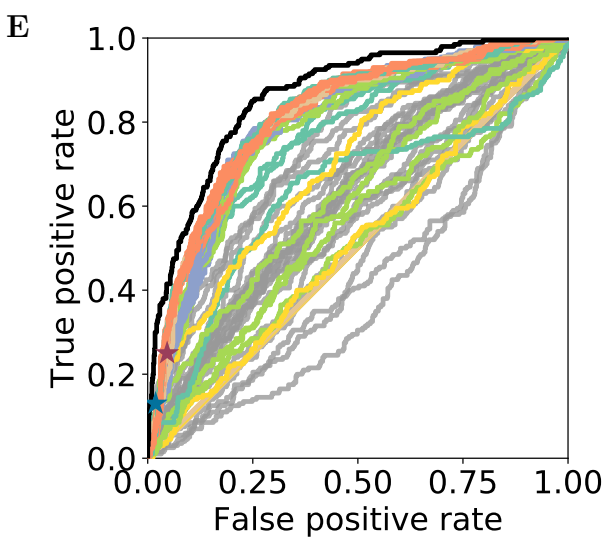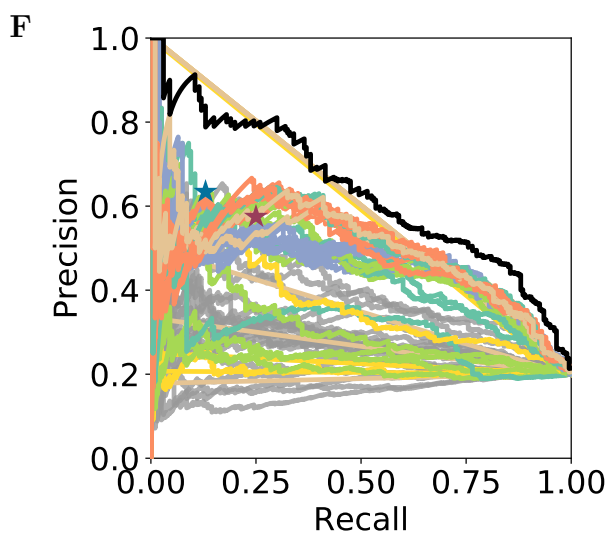

Supplement: Figure S1 [file rsos181806supp1.pdf]

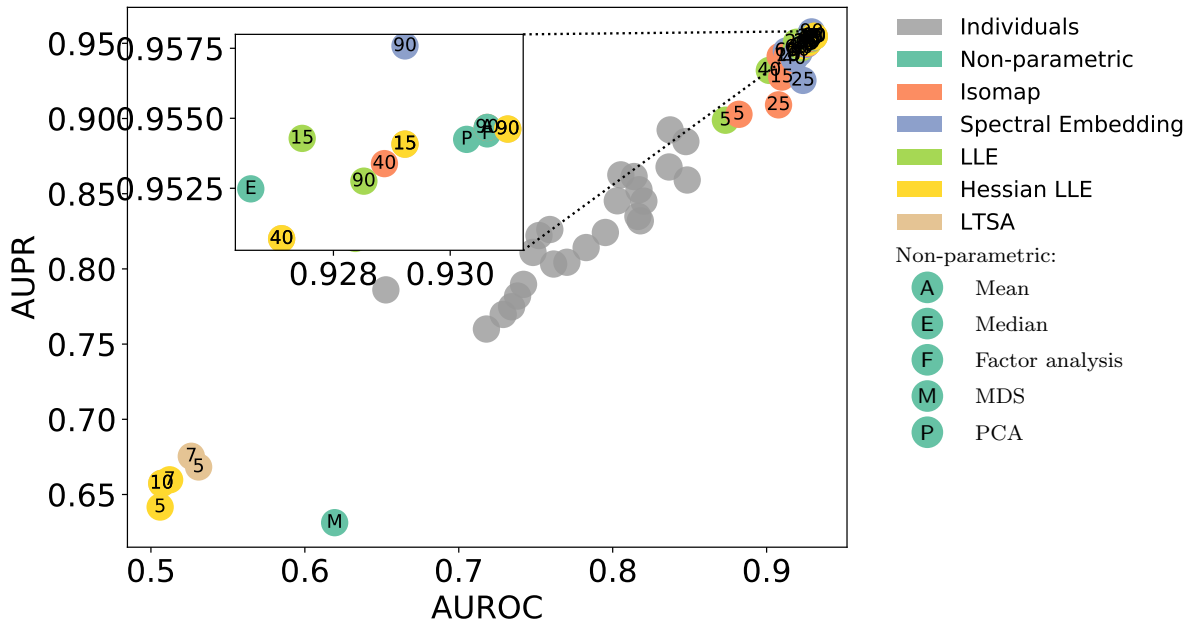

Supplement: Figure S2 [file rsos181806supp2.pdf]

A

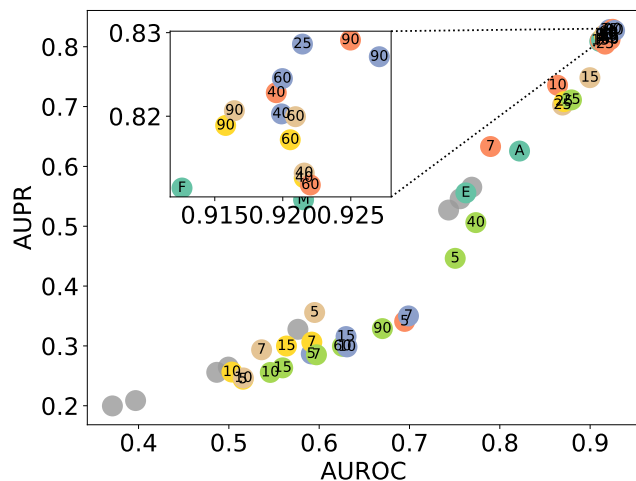

B

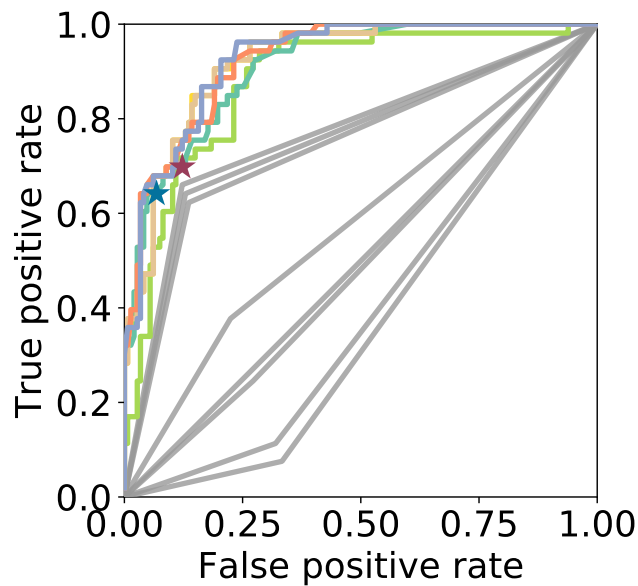

C

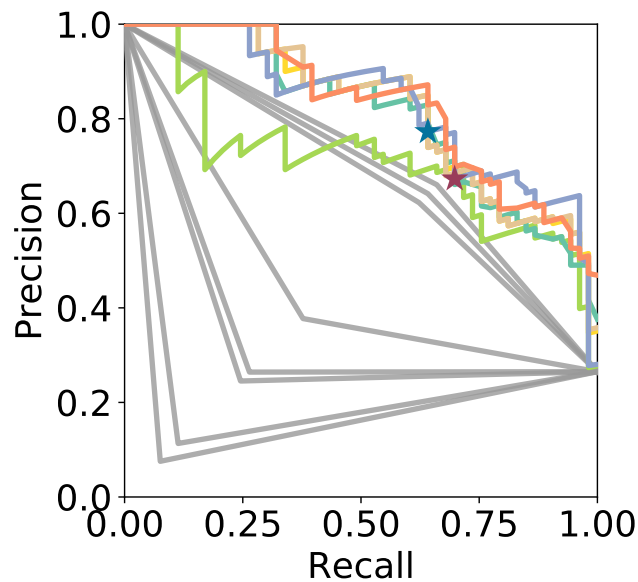

Supplement: Figure S3 [file rsos181806supp3.pdf]

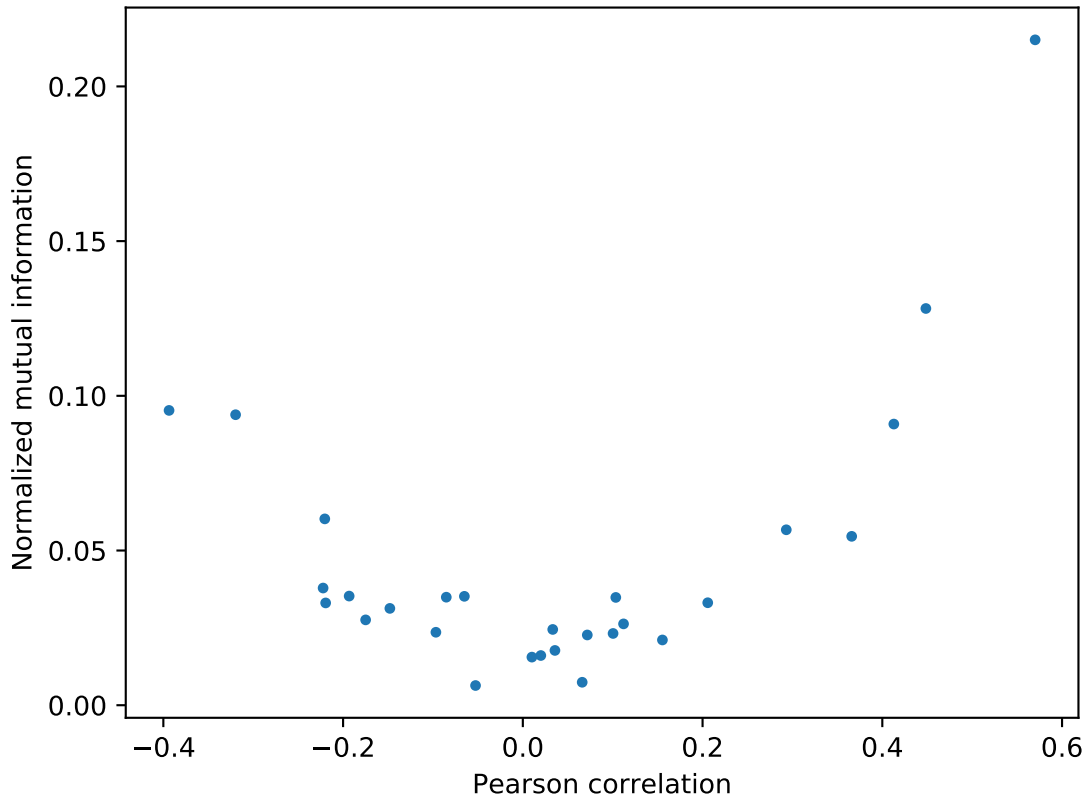

Supplement: Figure S4 [file rsos181806supp4.pdf]

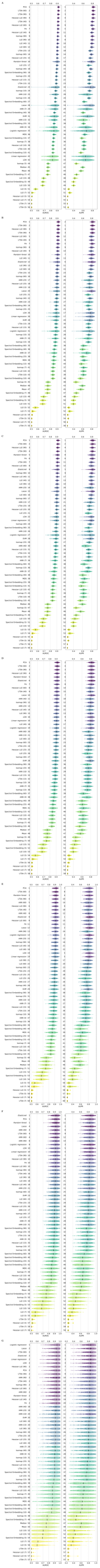

Supplement: Figure S5 [file rsos181806supp5.pdf]

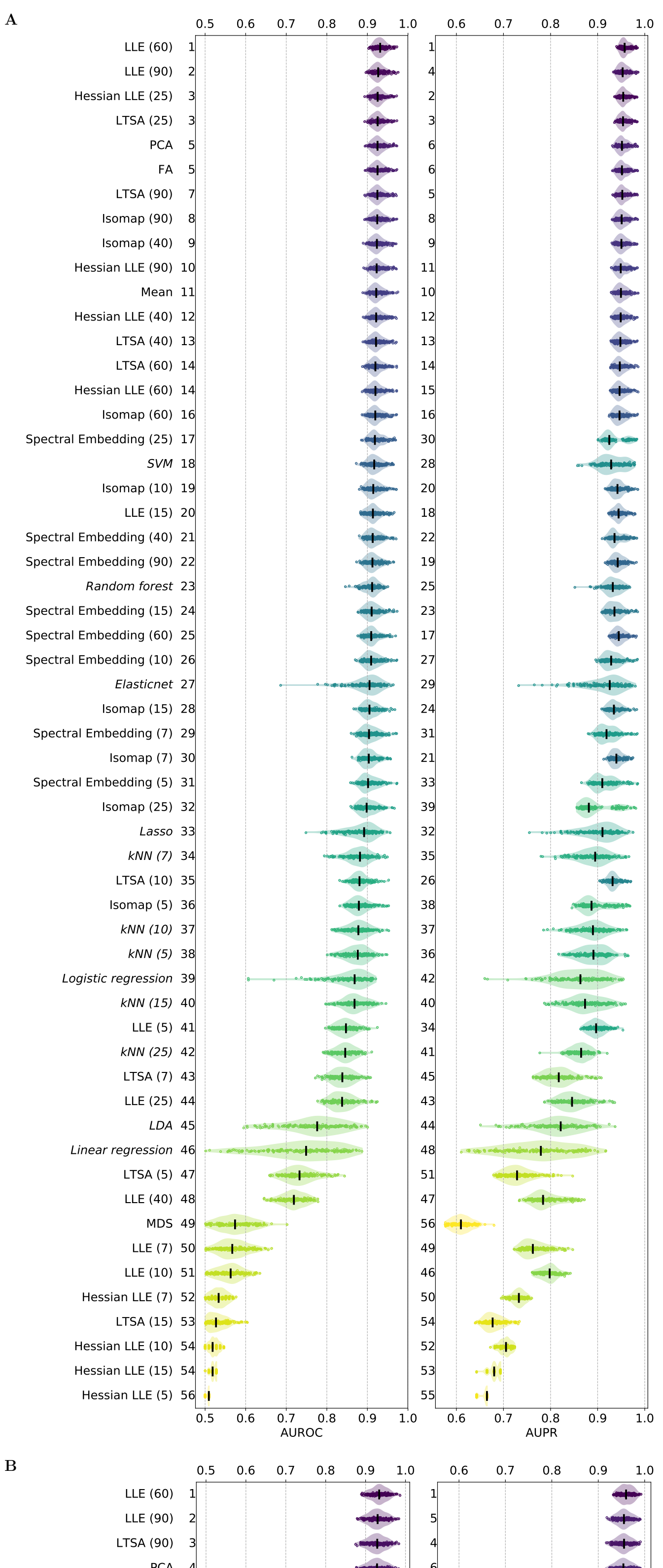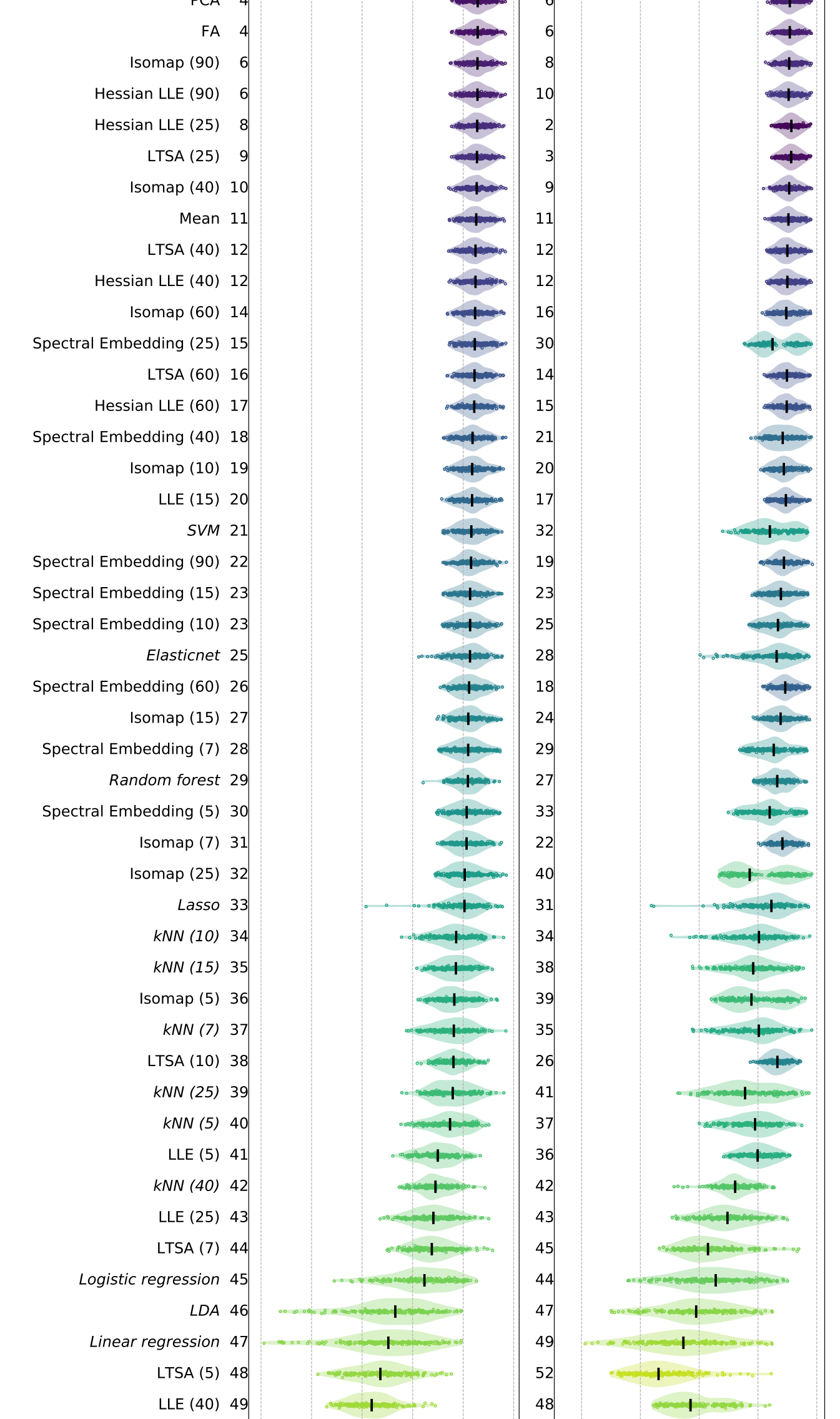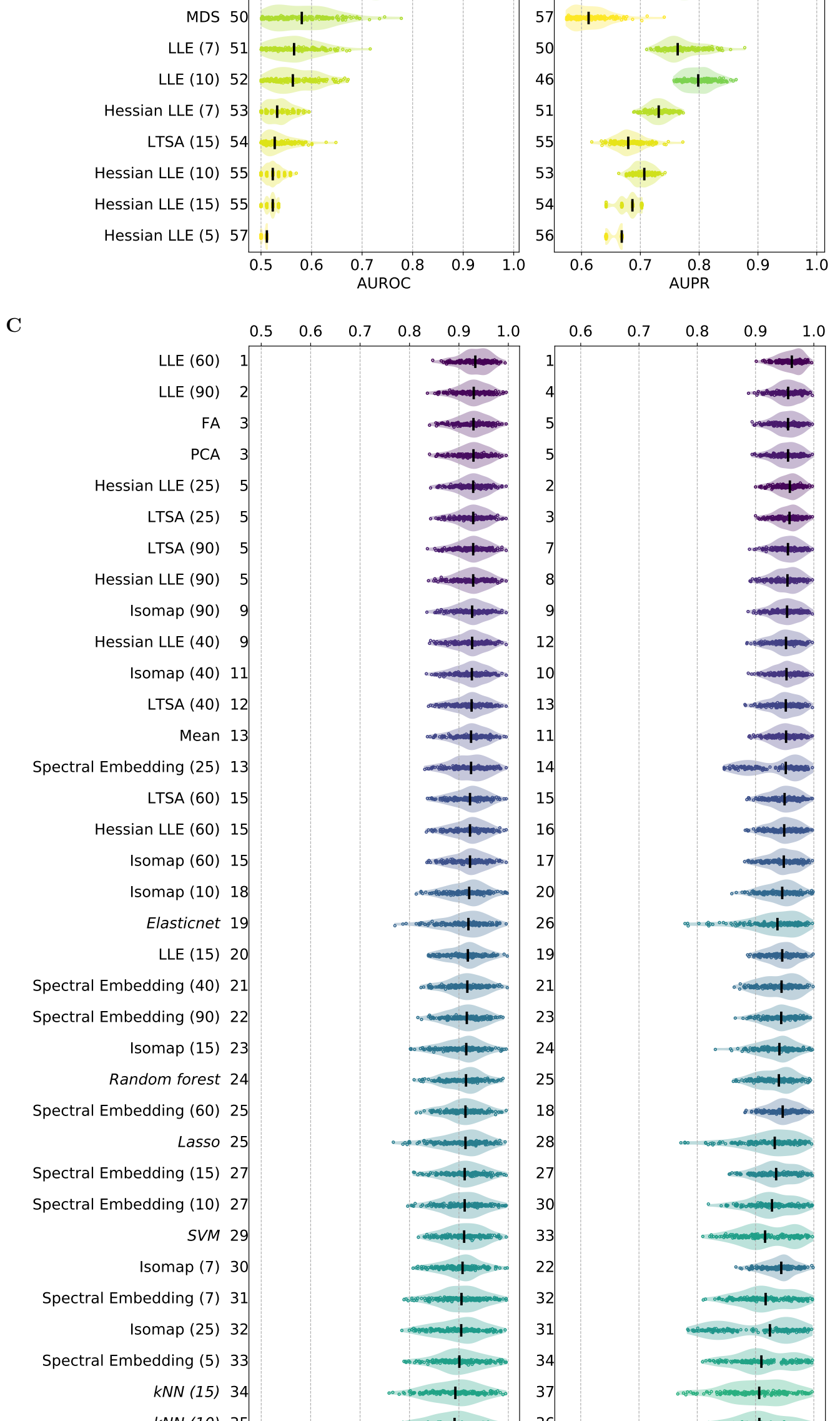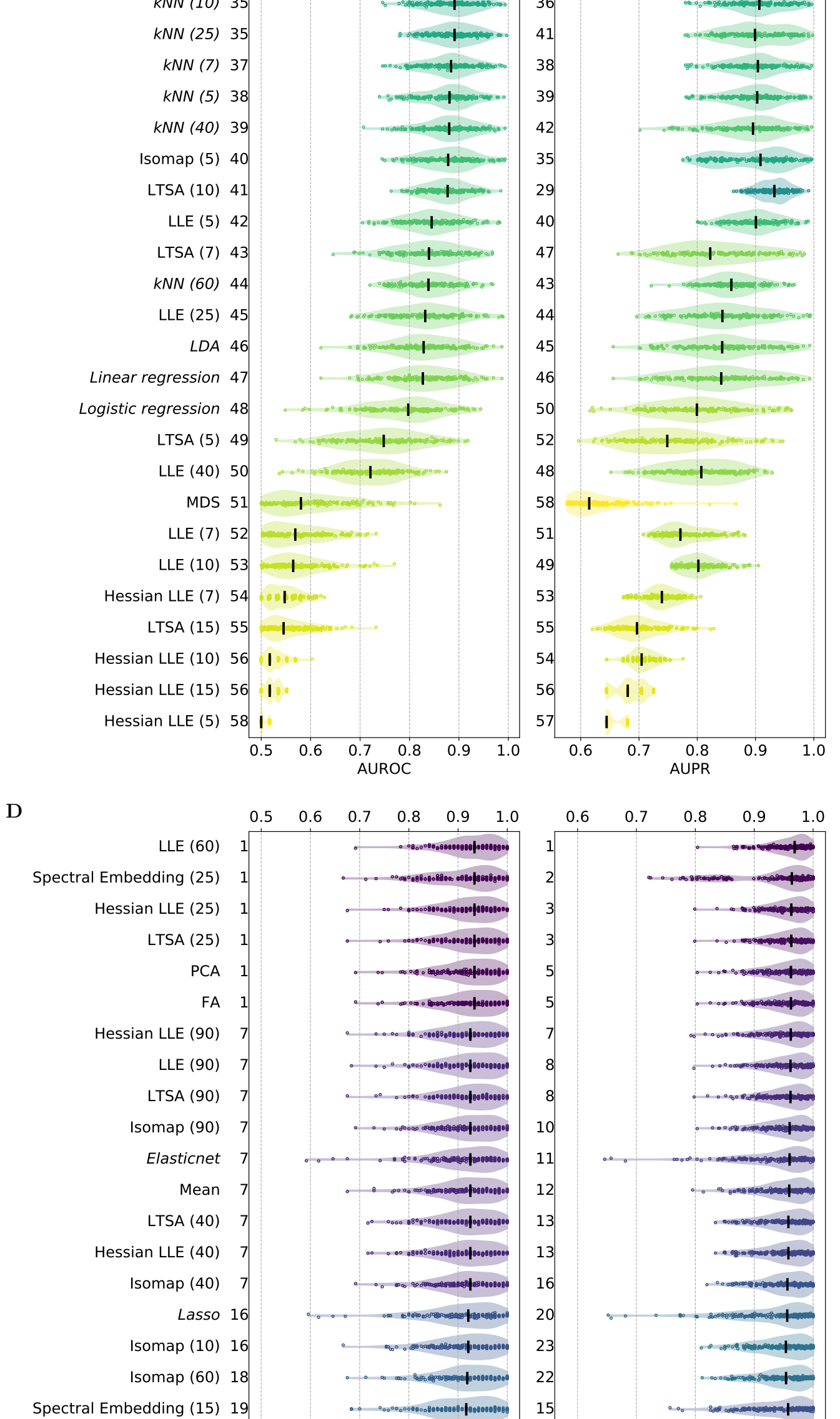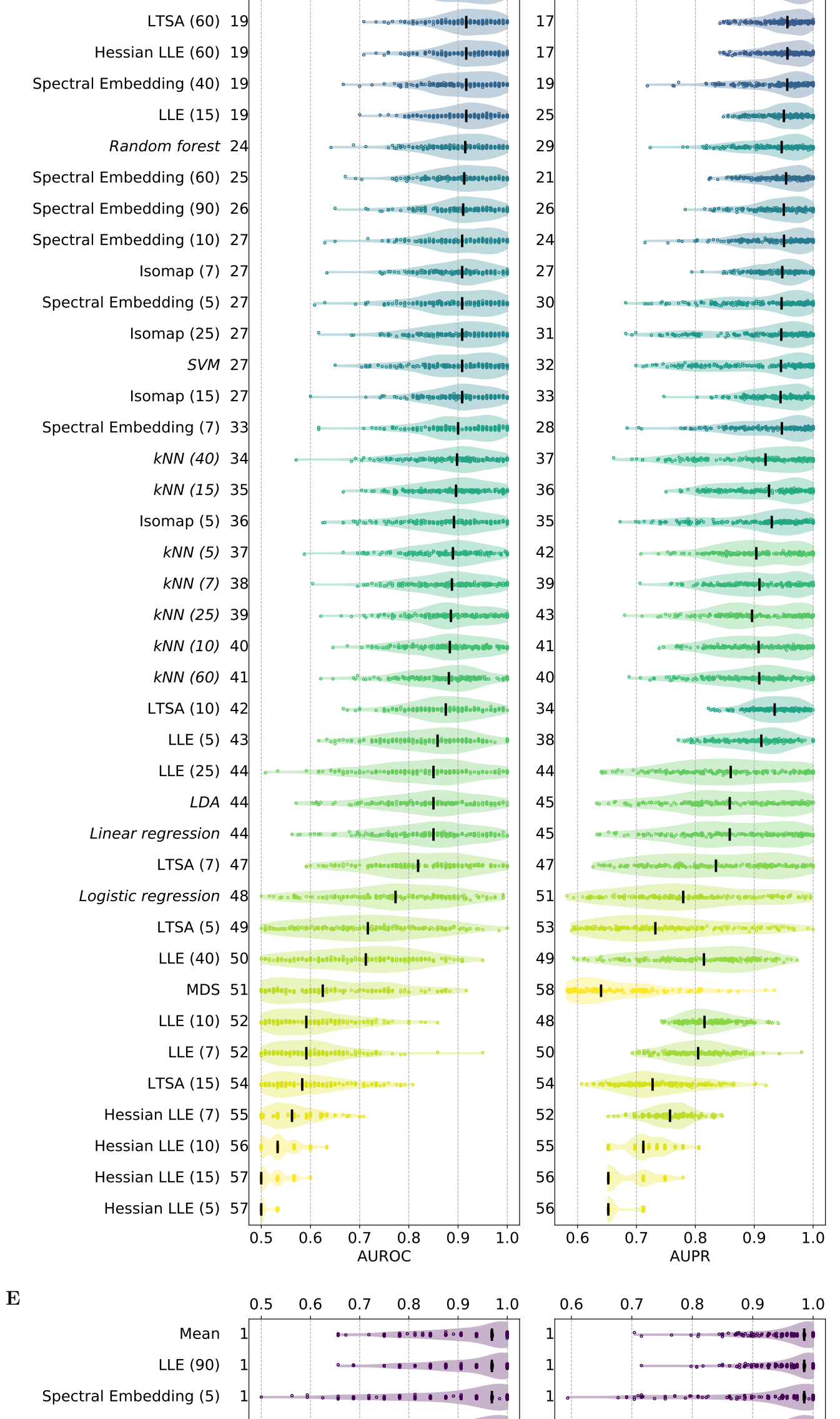

Supplement: Figure S6 [file rsos181806supp6.pdf]

A

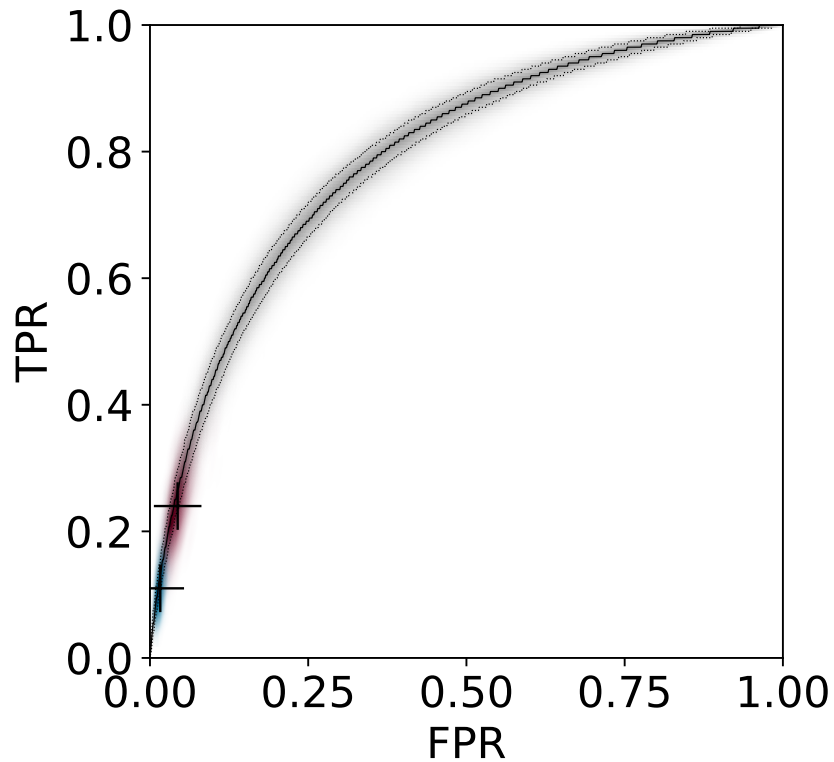

B

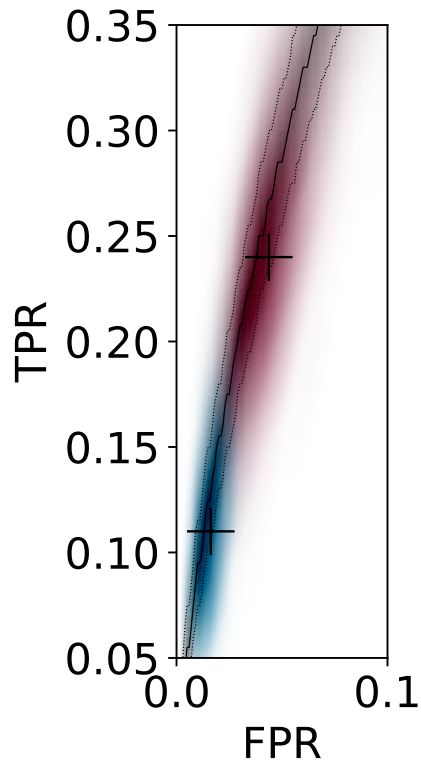

Supplement: Figure S7 [file rsos181806supp7.pdf]

Proportion of differences

$10^{-2}$

$10^{-3}$

$10^2$

$10^3$

Number of questions

- PCA v.s. SML
- PCA v.s. CUBAM

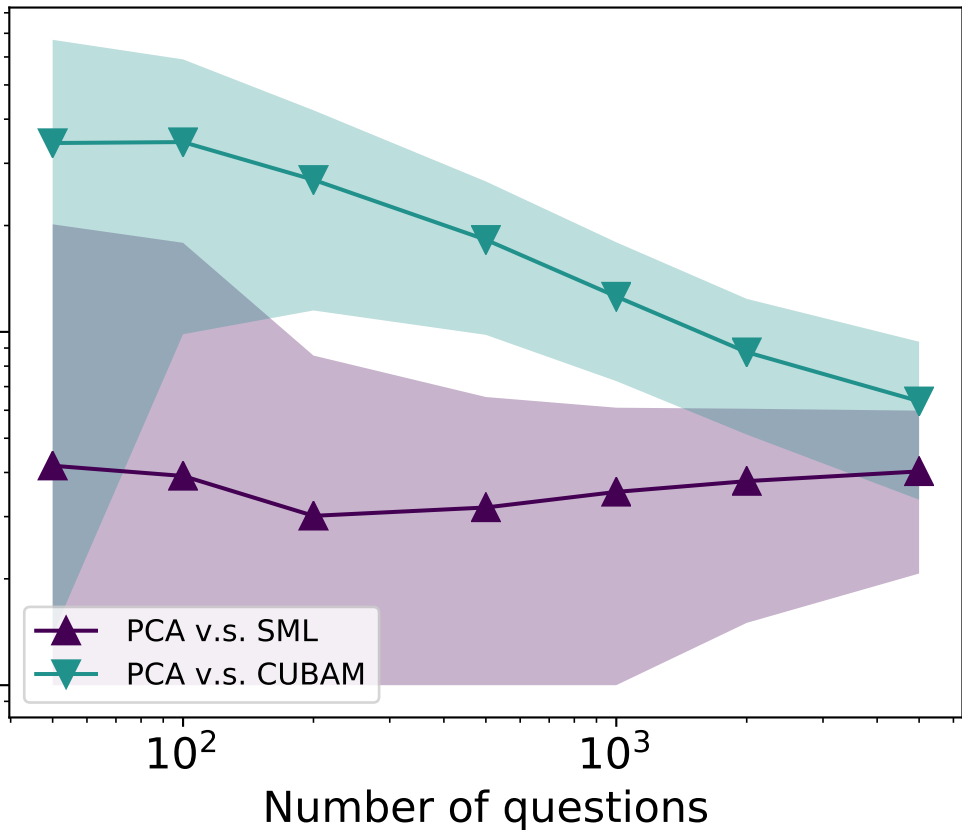

Supplement: Figure S8 [file rsos181806supp8.pdf]

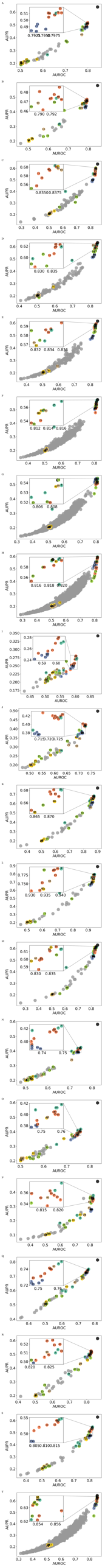

Supplement: Figure S9 [file rsos181806supp9.pdf]

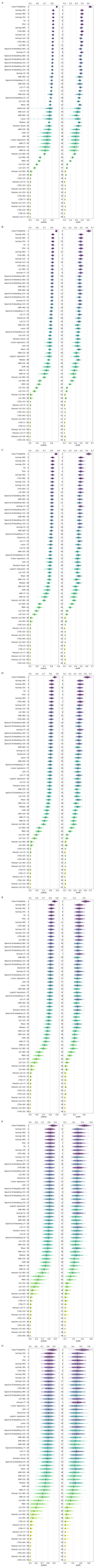

Supplement: Figure S11 [file rsos181806supp11.pdf]
